# Possible association of norepinephrine transporter -3081(A/T) polymorphism with methylphenidate response in attention deficit hyperactivity disorder

**DOI:** 10.1186/1744-9081-6-57

**Published:** 2010-10-07

**Authors:** Boong-Nyun Kim, Jae-Won Kim, Soon Beom Hong, Soo-Churl Cho, Min-Sup Shin, Hee-Jeong Yoo

**Affiliations:** 1 Division of Child and Adolescent Psychiatry, Department of Psychiatry, Seoul National University College of Medicine, Seoul, Republic of Korea

## Abstract

**Background:**

Attention-deficit/hyperactivity disorder (ADHD) is a heritable disorder characterized by symptoms of inattention and/or hyperactivity/impulsivity. Methylphenidate (MPH) has been shown to block the norepinephrine transporter (NET), and genetic investigations have demonstrated that the norepinephrine transporter gene (*SLC6A2*) is associated with ADHD. The aims of this study were to examine the association of the *SLC6A2 *-3081(A/T) and G1287A polymorphisms with MPH response in ADHD.

**Methods:**

This study enrolled 112 children and adolescents with ADHD. A response criterion was defined based on the Clinical Global Impression-Improvement (CGI-I) score, and the ADHD Rating Scale-IV (ARS) score was also assessed at baseline and 8 weeks after MPH treatment.

**Results:**

We found that the subjects who had the T allele as one of the alleles (A/T or T/T genotypes) at the -3081(A/T) polymorphism showed a better response to MPH treatment than those with the A/A genotype as measured by the CGI-I. We also found a trend towards a difference in the change of the total ARS scores and hyperactivity/impulsivity subscores between subjects with and without the T allele. No significant association was found between the genotypes of the *SLC6A2 *G1287A polymorphism and response to ADHD treatment.

**Conclusion:**

Our findings provide evidence for the involvement of the -3081(A/T) polymorphism of *SLC6A2 *in the modulation of the effectiveness of MPH treatment in ADHD.

## Background

Attention-deficit/hyperactivity disorder (ADHD) is a heritable neurodevelopmental disorder affecting about 3-7% of children with its symptoms of inattention and/or hyperactivity/impulsivity [1]. Methylphenidate (MPH) has been reported to reduce ADHD symptoms in approximately 70% of children with ADHD [2,3], and has been used for the treatment of the disorder for more than 60 years [4]. It has been well recognized that the mode of action of MPH in ADHD treatment is in its blockade of not only the dopamine transporter (DAT) [5] but also the norepinephrine transporter (NET) [6]. One recent study examined if MPH potentially blocked the human and mouse NET, and indeed reported the sensitivity of the NET to MPH as being similar to that of the DAT [7]. Andrews and Lavin [8] demonstrated that the MPH-induced increase in cortical cell excitability is mediated by activation of alpha-2-adrenergic receptors, and they suggested the possibility of the therapeutic actions of stimulants being associated with preferential activation of noradrenergic and/or dopaminergic neurotransmission within the prefrontal cortex.

In the prefrontal cortex, where DAT density is low and NET density is higher, it is unlikely that blockade of the DAT is a significant contributor to elevated DA levels in this region. It has been postulated that DA is transported by the NET in the prefrontal cortex, since DA has a higher affinity for the NET as compared with its affinity for the DAT [9]. DAT-selective (MPH), DAT/NET-selective (amphetamine), or NET-selective (atomoxetine) ADHD medications have one pharmacological effect in common, which is to elevate extracellular levels of DA and NE in the prefrontal cortex [10].

The norepinephrine transporter gene (*SLC6A2*), located on chromosome 16q12.2, is composed of 14 exons spanning 48 kb, predicting a protein of 617 amino acids. It has been suggested as one of the candidate genes associated with ADHD [11]. Recently, Kim *et al*. [12] demonstrated a -3081 A to T single nucleotide polymorphism (rs28386840) in the promoter region of the *SLC6A2 *and its association with ADHD. This study also identified the -3081(A/T) polymorphism as a functional polymorphism that decreases promoter function. According to a study by Yang *et al*. [13], the G1287A polymorphism (rs5569), at exon 9 of the *SLC6A2*, was identified to be associated with MPH response during the treatment of ADHD. Our previous study [14] provided evidence for the possible involvement of the *SLC6A2 *-3081(A/T) polymorphism in the expression of ADHD symptoms, such as increased variability in response time performance. However, in our study, the family-based and case-control association analyses of the -3081(A/T) and G1287A polymorphisms of the *SLC6A2 *found no significant association of these two polymorphisms with ADHD. Recently, the finding of no significant effect for *SLC6A2 *G1287A SNP was reproduced by McGough *et al*. [15].

Pharmacogenetic studies aim to identify genetic variations associated with drug treatment response or adverse effects secondary to medication [16,17]. There is growing interest in the pharmacogenetics of ADHD and, until recently, most of the investigations had focused on the potential susceptibility genes for ADHD, mainly the genes associated with the dopaminergic systems [18]. Neurobiological and pharmacological evidence points to dysregulation of the central noradrenergic systems as the underlying pathophysiology of ADHD [19]. The presumed mechanism of action of atomoxetine, which has shown clinical efficacy in treating ADHD patients, involves the selective norepinephrine reuptake inhibitor functions [20]. More precisely, atomoxetine is known to be more specific of the NET than the DAT, which stresses the rationale for the importance of further gene studies targeting the NET. This evidence supports the importance for a comprehensive assessment of the *SLC6A2 *in ADHD. In addition, the *SLC6A2 *is therefore also a likely candidate to assess genetic contributions to variability in ADHD treatment response. To our knowledge, most of the pharmacogenetic studies of *SLC6A2 *in ADHD have been conducted on Western populations [11,13,21], and there have been no studies on the association between the *SLC6A2 *-3081(A/T) polymorphism and the response to MPH treatment. The aims of this study were to examine the association of the *SLC6A2 *-3081(A/T) and G1287A polymorphisms with MPH response in children and adolescents with ADHD.

## Methods

### Subjects and clinical assessments

The participants of the present study were recruited from the Department of Child and Adolescent Psychiatry at Seoul National University Hospital in Korea. The diagnostic procedures in our unit have been described elsewhere [14]. In brief, ADHD was diagnosed based on the DSM-IV criteria using the Kiddie-Schedule for Affective Disorders and Schizophrenia-Present and Lifetime Version (K-SADS-PL) [22,23]. Subjects with (1) a history of, or a current neurological disease, including convulsive disorder, or (2) any evidence of a comorbid psychiatric condition, such as Tourette's disorder, mental retardation, pervasive developmental disorder, bipolar disorder, psychosis, language difficulties or learning disabilities, were excluded. Of the DSM-IV subtypes of ADHD, the combined subtype was the most common in our subjects (61.6%), followed by the inattentive (25.9%) and hyperactive-impulsive (5.4%) subtypes. With regard to comorbidity, oppositional defiant disorder (13.4%) was the most common, followed by anxiety disorder (10.7%) and enuresis (4.5%). For clinical evaluation of ADHD subjects, the ADHD Rating Scale-IV (ARS) [24,25] and Clinical Global Impression (CGI) [26] were administered. The ARS scores were checked by parents, and CGI scores were checked by psychiatrists.

The study was approved by the institutional review board (IRB) for human subjects at the Seoul National University Hospital. Parents provided written informed consent, and the children or adolescents provided verbal assent regarding participation in this study.

### MPH administration and treatment response

All of the ADHD subjects were administered MPH for a total of 8 weeks. We adjusted the MPH doses at the 2nd and the 4th week. The dosages were increased until doses were reached that were sufficient to achieve therapeutic effect, on the basis of the parents' reports of symptom improvement and side effects, and then these doses were maintained for the remaining of 8 weeks. Following Cheon *et al*. [27,28], clinical assessments were performed by psychiatrists at baseline prior to medication and after 8 weeks of MPH treatment in order to assess the improvement of ADHD symptoms. A "good" response was defined *a priori *as a CGI-I score of 1 or 2 points after MPH treatment, whereas a "poor" response was defined as a CGI-I score in the range of 3-7 points; the dichotomous response criterion was our primary outcome measurement [28,29]. We established strong inter-rater reliability before the start of medication (kappa = 0.89). Psychiatrists were blind to patients' genotypes.

### Genotyping

Genomic DNA was extracted from whole blood lymphocytes using a G-DEX TM II Genomic DNA Extraction Kit (Intron, Korea). The detection of a single nucleotide polymorphism was based upon analysis of primer extension products generated from previously amplified genomic DNA, using a chip-based matrix-assisted laser desorption/ionization time-of-flight (MALDI-TOF) mass spectrometry platform (Sequenom, California, USA). The *SLC6A2 *polymorphisms were genotyped as previously described [12,13], with slight modifications. In brief, oligonucleotide primers [5'-ACG TTG GAT GAG ACC CTA ATT CCT GCA CCC, and 5'-ACG TTG GAT GTT CAG GAC CTG GAA GTC ATC for the G1287A polymorphism; 5'-ACG TTG GAT GGT TTT CTT GCC CCT CAA GTG, and 5'-ACG TTG GAT GAG GGA AGG AAA CCA GGA GAA for the -3081(A/T) polymorphism] were used to generate polymerase chain reaction (PCR) products. The PCR was performed in a volume of 5 μl containing 1 × PCR buffer (TAKARA, Japan), 2.5 mM MgCl2, 0.2 mM of each dNTP, 0.1 U HotStar Taq Polymerase (Qiagen, Germany), 8 pM of each primer, and 4.0 ng of genomic DNA. The reaction consisted of denaturation at 95°C for 15 min, followed by 45 cycles at 95°C for 20 sec, 56°C for 30 sec, and 72°C for 1 min, with a final extension at 72°C for 3 min. Following the PCR, unincorporated dNTP was removed by the addition of 0.3 U of shrimp alkaline phosphatase and incubation for 20 min at 37°C, followed by 5 min at 85°C for enzyme inactivation. The total volume of each reaction was 9 μl, including hME enzyme (Thermo Sequenase, GE Healthcare, UK), ACT termination mix, and 5 uM of extension primer. The primer extension protocol was started at 94°C for 2 min, followed by 55 cycles at 94°C for 5 sec, 52°C for 5 sec, and 72°C for 5 sec. After desalting of the reaction products with SpectroCLEAN (Sequenom), samples were analyzed in the fully automated mode with the MALDI-TOF MassARRAY system (Bruker-Sequenom, California, USA). We used blank and negative control for each genotyping plate. For quality control of genotyping data, duplicate testing of 10% (11 samples) of randomly selected samples was performed in a blind manner. No discrepancies were found.

### Statistical analysis

Allele frequencies were estimated by counting, and the Hardy-Weinberg equilibrium was calculated based on these allele frequencies, using the goodness-of-fit χ^2 ^test. The estimation of allele frequencies and the test for the Hardy-Weinberg equilibrium were conducted for the genotypes of all subjects.

Group differences in the clinical variables involving continuous data were computed using an independent two sample t-test or one-way analysis of variance (ANOVA). Between-group comparisons involving categorical data were assessed using the χ^2 ^test or Fisher's exact test. We used the ANOVA and t-test to assess correlation between the genotype of *SLC6A2 *and the change in the ARS scores in ADHD subjects after MPH treatment. Predictors of MPH response were tested using univariate analysis of variance with general linear model procedure: the dependent variable was the CGI-I score, and the fixed factors were gene, final MPH dose, and gene × dose interaction. Effect size estimates for MPH response were based on Cohen *f *^2^. The significance level was set at p = 0.05/2(SNPs)*2(outcome measures) = 0.01. Power analysis was performed using G*Power 3 (Heinrich-Heine-University, Dusseldorf, Germany).

## Results

### Demographic and clinical characteristics

One hundred and twelve ADHD subjects (mean age = 9.1 ± 2.1 years) were enrolled, consisting of 92 boys (82.1%) and 20 girls (17.9%) (Table 1). The average total IQ of the ADHD subjects was 107.4 ± 13.7. The average score of overall ADHD symptoms according to the ARS, as measured by the parents of the ADHD subjects, was 26.9 ± 10.3. No baseline differences were found between the responders and non-responders in their demographic and clinical characteristics, including the ARS scores at study entry.

**Table 1:** Demographic and clinical characteristics of subjects with ADHD

	ADHD (n = 112)	Responders (n = 62)	Non-responders (n = 50)	p-value
Age in yr, mean (SD)	9.1 (2.1)	9.1 (2.0)	9.0 (2.2)	0.79
Sex (M/F)	92/20	54/8	38/12	0.13
IQ, mean (SD)	107.4 (13.7)	107.8 (13.2)	106.9 (14.4)	0.73
ADHD subtypes				0.30
Combined	61.6%	58.1%	66.0%	
Inattentive	25.9%	25.8%	26.0%	
Hyperactive-impulsive	5.4%	4.8%	6.0%	
NOS	7.1%	11.3%	2.0%	
Comorbidity				
Oppositional defiant disorder	13.4%	8.0%	20.0%	0.07
Anxiety disorder	10.7%	9.7%	12.0%	0.69
Enuresis	4.5%	6.5%	2.0%	0.26
ARS baseline scores, mean (SD)				
Total	26.9 (10.3)	25.9 (11.1)	28.3 (8.8)	0.23
Inattentive	15.1 (5.8)	14.2 (5.9)	16.3 (5.6)	0.07
Hyperactivity/impulsivity	11.8 (6.0)	11.6 (6.3)	12.0 (5.7)	0.75
Dosage of MPH (mg/day), mean (SD)				
Baseline dose	19.9 (8.3)	18.6 (6.2)	21.5 (10.1)	0.08
Final 2 weeks dose	29.2 (11.6)	28.2 (10.9)	30.4 (12.4)	0.32

ADHD, Attention-Deficit/Hyperactivity Disorder; NOS, Not Otherwise Specified; ARS, ADHD Rating Scale; MPH, Methylphenidate

### Genetic polymorphisms of *SLC6A2*

Among the 112 ADHD subjects, the genotype frequencies (A/A homozygous, A/T heterozygous, and T/T homozygous) of the *SLC6A2 *-3081(A/T) polymorphism were 25.9%, 53.6%, and 20.5%, respectively (Table 2). The genotype frequencies (G/G homozygous, G/A heterozygous, and A/A homozygous) of the *SLC6A2 *G1287A polymorphism were 51.8%, 37.5%, and 10.7%, respectively. Genotype and allele frequencies observed in this study were comparable with previously reported values from the South Korean population [14]. The distribution of the genotypes for the *SLC6A2 *-3081(A/T) polymorphism and *SLC6A2 *G1287A polymorphism were in agreement with the expected values of the Hardy-Weinberg equilibrium (p > 0.05).

**Table 2:** Association between *SLC6A2 *genotypes and response to MPH treatment according to the CGI-I

		Response to MPH by CGI-I N (% within *SLC6A2 *genotype)			
				
	*SLC6A2 *genotype	Poor (CGI-I: 3-7)	Good (CGI-I: 1 or 2)	Total (% within total number)	p-value
-3081(A/T) polymorphism	A/A	18 (62.1%)	11 (37.9%)	29 (25.9%)	0.08
	A/T	24 (40.0%)	36 (60.0%)	60 (53.6%)	
	T/T	8 (34.8%)	15 (65.2%)	23 (20.5%)	
	A/A	18 (62.1%)	11 (37.9%)	29 (25.9%)	0.03
	A/T+T/T	32 (38.6%)	51 (61.4%)	83 (74.1%)	
	Total	50 (44.6%)	62 (55.4%)	112	
					
G1287A polymorphism	G/G	22 (37.9%)	36 (62.1%)	58 (51.8%)	0.12
	G/A	24 (57.9%)	18 (42.9%)	42 (37.5%)	
	A/A	4 (33.3%)	8 (66.7%)	12 (10.7%)	
	G/G	22 (37.9%)	36 (62.1%)	58 (51.8%)	0.14
	G/A+A/A	28 (51.9%)	26 (48.1%)	54 (48.2%)	
	Total	50 (44.6%)	62 (55.4%)	112	

### Association between the genotypes of *SLC6A2 *and MPH response according to the CGI-I assessed by the clinician

There was a **trend for **association between the genotypes of the *SLC6A2 *-3081(A/T) polymorphism and response to ADHD treatment. Of the subjects who had the T allele as one of the alleles (A/T or T/T genotypes) at the -3081(A/T) polymorphism, 61.4% (51 of 83) showed a good response (CGI-I = 1 or 2) to MPH treatment. However, only 37.9% (11 of 29) of the subjects with the A/A genotype showed a good response to MPH treatment (Pearson χ^2 ^(1) = 4.81, p = 0.03) (Table 2). The power to detect differences at the 0.01 level of significance with our sample size of 112 was 0.35. No significant association was found between the genotypes of the *SLC6A2 *G1287A polymorphism and response to ADHD treatment.

We found no significant gene effect (*F*_2,80 _= 0.49, p = 0.61, *f *^2 ^= 0.01) or gene × dose interaction (*F*_13,80 _= 1.19, p = 0.30, *f *^2 ^= 0.19) for the *SLC6A2 *-3081(A/T) polymorphism on MPH response.

### Association between the genotypes of the -3081(A/T) polymorphism and MPH response according to the ARS as assessed by the parents

There were no significant differences in the demographic and clinical characteristics, except the profile of comorbid enuresis, between the ADHD subjects with the (A/T + T/T) genotypes and those with the A/A genotype of the -3081(A/T) polymorphism (Table 3).

**Table 3:** Demographic and clinical characteristics of ADHD subjects according to genotypes of the -3081(A/T) polymorphism

	ADHD subjects with A/A genotype	ADHD subjects with A/T + T/T genotypes	p-value
Age in yr, mean (SD)	9.0 (2.4)	9.1 (2.0)	0.81
Sex (M/F)	22/7	70/13	0.31
IQ, mean (SD)	107.2 (13.3)	107.5 (14.0)	0.92
ADHD subtypes			0.35
Combined	58.6%	62.7%	
Inattentive	34.5%	22.9%	
Hyperactive-impulsive	0.0%	7.2%	
NOS	6.9%	7.2%	
Comorbidity			
Oppositional defiant disorder	6.9%	15.7%	0.19
Anxiety disorder	3.4%	13.3%	0.13
Enuresis	13.8%	1.2%	0.02
ARS baseline scores, mean (SD)			
Total	23.9 (11.4)	27.9 (9.7)	0.08
Inattentive	13.6 (6.3)	15.6 (5.6)	0.12
Hyperactivity/impulsivity	10.3 (6.9)	12.3 (5.7)	0.14
Dosage of MPH (mg/day), mean (SD)			
Baseline dose	19.7 (9.3)	20.0 (8.0)	0.88
Final 2 weeks dose	27.9 (11.1)	29.7 (11.8)	0.48
CGI-I score, mean (SD)	2.8 (0.9)	2.4 (0.8)	0.04

ADHD, Attention-Deficit/Hyperactivity Disorder; NOS, Not Otherwise Specified; ARS, ADHD Rating Scale; MPH, Methylphenidate

When we compared the changes in ARS scores after MPH treatment as the secondary outcome measurement, according to the genotypes of the -3081(A/T) polymorphism, we found a trend towards a difference in the change of the total ARS scores [t(1) = 1.92, p = 0.06] and hyperactivity/impulsivity subscores [t(1) = 1.73, p = 0.09] between subjects with and without the T allele (Table 4).

**Table 4:** Comparison reductions in ARS scores after MPH treatment in ADHD subjects according to genotypes of the -3081(A/T) polymorphism

Genotype	Changes in ARS scores					
	
	IA	p-value	Hy/Imp	p-value	Total	p-value
A/A	5.4 (4.3)	0.54	4.6 (4.1)	0.12	8.4 (7.6)	0.10
A/T	6.2 (4.9)		6.3 (4.5)		12.1 (8.8)	
T/T	7.2 (6.6)		7.8 (7.2)		14.7 (12.6)	
A/A	5.4 (4.3)	0.38	4.6 (4.1)	0.09	8.4 (7.6)	0.06
A/T + T/T	6.5 (5.4)		6.7 (5.4)		12.8 (10.0)	

ARS, ADHD Rating Scale; IA, inattentive subscale; Hy/Imp, hyperactivity/impulsivity subscale

All values are mean (± S.D.)

## Discussion

In this study, we identified a trend for association between the -3081(A/T) polymorphism of *SLC6A2 *and response to MPH treatment in Korean children and adolescents with ADHD. Those ADHD subjects who had the T allele as one of the alleles (A/T or T/T genotypes) at the -3081(A/T) polymorphism showed a better response to MPH treatment than those with the A/A genotype, although this relative difference does not provide definite conclusion on whether the presence of the T allele is association with a better response or the absence of the T allele is associated with a poorer response, given that even the ADHD patients with the T allele showed only a 61.4% response rate to MPH. In addition, the ADHD subjects with the T allele showed a tendency for more symptom reduction after treatment with MPH than those without the T allele. However, no significant association was found between response to MPH and the *SLC6A2 *G1287A polymorphism. In a previous study [13], those ADHD subjects who were homozygous for the A allele (A/A genotype) at the G1287A polymorphism showed less symptom reduction in the hyperactive-impulsive subscores of the ARS after treatment with MPH than those with the other genotypes (G/G or G/A genotypes). The sample size of their study (35 boys and 10 girls) was smaller than that of our study (92 boys and 10 girls). The divergent results between the two studies might reflect methodological issues, such as sample sizes, ethnic differences, inclusion and exclusion criteria, or instruments to assess drug treatment response.

It is important to understand the potential functional significance of the *SLC6A2 *-3081(A/T) polymorphism. Kim *et al*. [12] reported that the -3081(T) allele significantly decreases promoter function compared with the -3081(A) allele, which was assessed using synthesized promoter-reporter constructs. The authors also demonstrated that Slug and Scratch, neural-expressed transcriptional repressors, decrease the promoter activity only when it contains the -3081(T) allele. In their study, the frequency of the -3081(T) allele was significantly higher in the ADHD probands than in the controls, and the A/T and T/T genotypes were overrepresented in the ADHD subjects. However, studies of the association between *SLC6A2 *-3081(A/T) polymorphism and ADHD have yielded mixed results, with several studies finding some evidence for association [12,27], and our previous paper providing no evidence for association [14]. Recently, Jung *et al*. [30] reproduced in a Korean population that the frequency of the -3081(T) allele was significantly higher in ADHD subjects than in controls. Our current data that the ARS total score at baseline showed a higher trend (p = 0.08) in those with at least one -3081(T) allele might slightly support the previous findings from the Korean population. Downregulated promoter function of *SLC6A2 *and consequent decrease in transcriptional activity, as reported by Kim *et al*. [12], may result in low levels of NET. Our results, which suggest a good response to MPH in ADHD is associated with the presence of the -3081(T) allele of *SLC6A2*, may be explained by reduced levels of NET within the brain. On the other hand, it is possible that subjects with the -3081(T) allele have their ADHD in tighter relation with the action of NET, and therefore pharmacologically blocking this transporter is associated with relatively better treatment response. Thus, investigating response to treatment and its mechanism of action in terms of molecular and genetic findings might help us to identify more homogeneous subgroups of ADHD [31]. Further studies using imaging genetic approaches based on single photon emission computerized tomography (SPECT) or positron emission tomography (PET) will be required to investigate NE gene effects on regional cerebral perfusion or metabolism in ADHD and to evaluate the association of *SLC6A2 *genetic variation with levels of NE activity in the brain.

The search for candidate genes associated with ADHD has been largely driven by the understanding that medications for this disorder have drug targets in the catecholamine neurotransmitter systems [32]. Although knowledge about the presumed mechanisms of action of ADHD medication, including MPH, initially informed the research into genetic polymorphisms associated with the disorder, these same polymorphisms have been and continue to be logical candidates to predict medication outcome, in terms of symptom response and side effect profiles [33]. Recent studies suggest that candidate genes involved in catecholamine pathways influence individual responses to ADHD treatments. However, as mentioned above, the majority of pharmacogenetic studies of ADHD investigating response or tolerability to medication have focused mainly on dopaminergic genes [11]. Polymorphisms in noradrenergic genes, such as alpha-2A-adrenergic receptor gene (*ADRA2A*) or *SLC6A2*, may have a specific effect as proposed on MPH response [13,21,34]. Mick *et al*. [21] have conducted a genome-wide association study (GWAS) on a sample of 187 ADHD children and found that 2 SNPs that tag NET gene (*SLC6A2*) were suggestively association with MPH response. On the other hand, in a study conducted by Kooij *et al*. [35], the polymorphisms in the *SLC6A2 *were not associated with MPH response. Here, of note is that the study by Ramoz *et al*. [11] has demonstrated that the *SLC6A2 *predicted response to another pharmacological agent for ADHD: atomoxetine. In these contexts, our findings demonstrate that the -3081(A/T) polymorphism of *SLC6A2 *might modulate the effectiveness of MPH treatment on ADHD. To date, this study is the first to examine the association of the *SLC6A2 *-3081(A/T) polymorphism with MPH response in ADHD. The result of Ramoz *et al*. [11] and ours combined further suggest that the *SLC6A2 *-3081(A/T) polymorphism constitute a common pathway for the treatment effects of both MPH and atomoxetine.

Several limitations to this study should be noted. First, this was a naturalistic study, and we did not have a placebo arm in this trial. It is likely that a placebo response in our study group would have decreased the statistical power by reducing the measurement precision of MPH response. However, naturalistic study designs may be valuable to better appreciate the role of genetic factors in routine clinical practice beyond the realm of controlled clinical trials. Second, our study population included all of the subtypes of ADHD, which might have contributed to clinical heterogeneity; the subtypes may have acted as potential confounders of the investigated association. Third, MPH was administered with no control of adherence by investigators. Fourth, we did not control for the two types of MPH products: immediate-release (IR) MPH and sustained-release (SR) MPH. However, we did not find a significant difference in clinical improvement between the ADHD subjects treated with IR MPH and those treated with SR MPH (data not shown, but available upon request). Lastly, but perhaps most importantly, two titration visits in our study design were probably insufficient, which may have resulted in lower mean daily doses at endpoint, and it might explain the low response rate observed in our results compared to the reported average response rate of approximately 75% in controlled outpatient stimulant trials [36].

## Conclusion

In conclusion, our data suggest that the -3081(A/T) polymorphism of *SLC6A2 *might be involved in the modulation of the effectiveness of MPH treatment in ADHD. Further pharmacogenetic investigations should expand the focus to include other functional polymorphisms of the *SLC6A2*, given the increased use of noradrenergic drugs in the treatment of ADHD symptoms [37], in order to better understand the role of genetic variation in a good *vs*. poor response to drug treatment for ADHD.

## Competing interests

The authors declare that they have no competing interests.

## Authors' contributions

BNK, JWK, SCC and HJY designed the study and participated in data collection. BNK, JWK and SBH analyzed the data, interpreted the results, and drafted the manuscript. SCC and MSS supervised the study. All authors read and approved the final manuscript.
